# In vitro assessment of iron availability from commercial Young Child Formulae supplemented with prebiotics

**DOI:** 10.1007/s00394-016-1353-3

**Published:** 2016-12-09

**Authors:** Tatiana Christides, Julia Clark Ganis, Paul Anthony Sharp

**Affiliations:** 1 Department of Life and Sports Sciences, Faculty of Engineering and Science, University of Greenwich, Medway Campus, Chatham Maritime, Kent ME4 4TB UK; 2 Metal Metabolism Group, Diabetes and Nutritional Sciences Division, School of Medicine, King’s College London, London, UK

**Keywords:** Iron, Bioavailability, Prebiotics, Young children, Young Child Formulae, Caco-2

## Abstract

**Purpose:**

Iron is essential for development and growth in young children; unfortunately, iron deficiency (ID) is a significant public health problem in this population. Young Child Formulae (YCF), milk-derived products fortified with iron and ascorbic acid (AA, an enhancer of iron absorption) may be good sources of iron to help prevent ID. Furthermore, some YCF are supplemented with prebiotics, non-digestible carbohydrates suggested to enhance iron bioavailability. The aim of our study was to evaluate iron bioavailability of YCF relative to prebiotic and AA concentrations. We hypothesised that YCF with the highest levels of prebiotics and AA would have the most bioavailable iron.

**Methods:**

We used the in vitro digestion/Caco-2 cell model to measure iron bioavailability from 4 commercially available YCF with approximately equal amounts of iron, but varying amounts of: AA and the prebiotics fructo- and galacto-oligosaccharides. Caco-2 cell ferritin formation was used as a surrogate marker for iron bioavailability.

**Results:**

The YCF with the highest concentration of prebiotics and AA had the highest iron bioavailability; conversely, the YCF with the lowest concentration of prebiotics and AA had the lowest. After the addition of exogenous prebiotics, so that all tested YCF had equivalent amounts, there was no longer a significant difference between YCF iron bioavailability.

**Conclusion:**

Our results suggest that ascorbic acid and prebiotics in YCF improve iron bioavailability. Ensuring that iron is delivered in a bioavailable form would improve the nutritional benefits of YCF in relation to ID/IDA amongst young children; therefore, further exploration of our findings in vivo is warranted.

## Introduction

Iron is essential for growth and neurodevelopment in infants and young children (children between the ages of 1–3). Humans from birth until 3 years of age undergo rapid brain growth and development; iron is required for multiple processes underlying this developmental surge including myelination, neurotransmitter function, brain energy homoeostasis, and neuronal and synaptogenesis [1]. Inadequate iron during this period of childhood is associated with impaired cognitive and psychomotor development that may not reverse even if children become iron-replete later in life [2]. Full-term infants are protected from iron deficiency and iron deficiency anaemia (ID/IDA) for the first 4–6 months after birth as a result of gestationally derived iron stores [3]. Unfortunately, for the rest of the critical time period up to 3 years of age, children are at high risk for ID/IDA because of rapid overall growth and associated increased iron needs and often inadequate intake of bioavailable dietary iron [3, 4].

Infant formula is fortified with iron to prevent deficiency [5], and the evidence suggests that this mandatory fortification is effective—in the developed world less than 5% of infants under 12 months have IDA [3, 6]. However, the prevalence of ID/IDA increases between 1–3 years of age, ranging from 6 to 15% in the developed world [7, 8], due to accelerated growth at this developmental stage, and also possibly the switch from formula to cow’s milk that has less iron compared with formula [9–11]. In response, the food industry has developed “Young Child Formulae” (YCF), milk-derived products fortified with multiple micronutrients and other dietary factors, aimed at 1- to 3-year-old children. YCF contain more iron compared with cow’s milk and are also fortified with ascorbic acid, a known enhancer of non-haem iron absorption.

There are numerous commercially available YCF throughout the developed world. In the United Kingdom (UK), according to the Diet and Nutrition Survey of Infants and Young children, 18% of children between 12 and 18 months received YCF as part of their diet, representing a significant number of children consuming these products [12]. Furthermore, a cross-sectional study of 1- to 2-year-old children conducted in France found that use of YCF was associated with improved dietary iron intake compared with children exclusively receiving cow’s milk [10]; however, the specific nutrient composition of the YCF being consumed with reference to modifiers of iron absorption was not specified, nor were iron status markers measured in study subjects to assess whether the observed increase in iron intake correlated with good iron status.

In addition to added iron and ascorbic acid, several YCF products are supplemented with prebiotics. Prebiotics are non-digestible dietary substances, typically oligosaccharides such as fructo- and galacto-oligosaccharides (FOS and GOS, respectively) that encourage the growth of beneficial microorganisms in the large intestine [13, 14]. Prebiotics have also been shown to improve the bioavailability of minerals, although results with regard to iron are not consistent [15]. The type and amount of added prebiotic varies between YCF, and some YCF do not have any added prebiotics (source of information: manufacturers’ nutrition labels and websites). Of note, The European Society for Pediatric Gastroenterology, Hepatology and Nutrition (ESPGHAN) Committee on Nutrition in a recent review found that there was no evidence of adverse effects from the addition of prebiotics to formula; however, they also concluded that evidence was insufficient to recommend routine supplementation and that further research was needed [16]. The European Commission regulates infant and follow-on formulae composition in the European Union and stipulates a maximum amount of prebiotics that may be added, but does not make more specific reference to recommended amounts (Commission Directive 2006/141/EC, reviewed and updated July 2016) [17].

Although young children are at increased risk for ID/IDA, there is also concern that excess dietary iron may affect their gut microbiome with negative local and systemic health effects [18]. A study conducted in Kenya found that intake of iron-fortified foods led to a higher number of gut pathogens, increased intestinal inflammation and more frequent diarrhoea episodes requiring treatment [19]. In another study, consumption of iron-fortified biscuits was associated with an unfavourably altered gut microbiome [20]. Unabsorbed iron reaching the colon is suggested to mediate these effects [21]. Of note, however, a study in children from 9 to 18 months living in the UK, who continued to receive iron supplemented formula, found no evidence of adverse effects over the 9 months of the study even in iron-replete children [22]; thus, the effects of iron on the young gut may vary depending on underlying risk for gut inflammation and infection. Nonetheless, foods fortified with highly bioavailable iron are desirable because of their efficacy in improving iron nutriture as a result of increased absorption. They may also, as a consequence of improved small bowel uptake, deliver less iron to the large intestine that may have long-term health implications related to the gut microbiome.

The objective of this study was to compare the iron bioavailability of four YCF commonly consumed in the UK containing approximately equal amounts of iron, but with different ascorbic acid concentrations, and varying amounts and types of FOS and GOS. The aim of our research was to evaluate YCF iron bioavailability relative to supplemental prebiotic and ascorbic acid concentrations. We utilised the Caco-2 cell/in vitro digestion model with the formation of Caco-2 cell ferritin as a surrogate marker for cellular iron uptake. This model has been used in multiple studies to assess iron bioavailability from foodstuffs including milks, milk-derived products and infant formulae, for examples see [23–25]. We hypothesised that the YCF with the highest concentrations of prebiotics and ascorbic acid would have the highest levels of bioavailable iron, while the YCF with no added prebiotics and lower levels of ascorbic acid would have the lowest iron availability.

## Materials and methods

### Reagents

All chemicals and enzymes, unless otherwise stated, were purchased from Sigma-Aldrich (UK). Acids required for glassware cleaning and in vitro digestions were acquired from VWR (UK). Thermo Fisher Scientific was the provider of cell culture media, flasks, tissue culture plates and cell culture reagents. Twenty-four hours prior to each experiment glass- and plastic-ware needed for experiments were soaked in 10% (v/v) trace metal grade 68% nitric acid and then subsequently rinsed with 18 mΩ pure water.

GOS powders (100% purity) used in experiments were either purchased from Megazyme International (Ireland), or kindly provided by King-Prebiotics. FOS powder (100% purity) was purchased from Health Plus (Seaford, East Sussex).

### Samples

Four YCF were obtained from three different leading UK supermarkets on three separate occasions, herein identified as YCF A, B, C and D. Products were tested before the use by date and within 1 month of purchase. YCF were stored unopened at room temperature, similar to their distribution and market environment. YCF A and B were fortified with equivalent amounts of prebiotics in the forms of GOS and FOS by the manufacturer; YCF A specified the ratio of GOS to FOS, but neither YCF A nor B provided nutrient label information on the specific forms of GOS and FOS used. YCF C was fortified with GOS alone by the manufacturer, and at a lower concentration compared with YCF A and B, and YCF D had no added prebiotics. YCF A, B and D had 1.2 mg iron/100 ml product; YCF C had 1.1 mg/100 ml. Ascorbic acid levels also differed between YCF; YCF A and B had the highest reported levels, followed by YCF D. YCF C had the lowest reported concentration of vitamin C. The nutrient composition of the various YCF in relation to total iron, ascorbic acid and prebiotics is provided below (Table 1).

**Table 1:** Nutrient composition of YCFs A–D according to the manufacturers’ labels and websites

Nutrient (mg/100 ml unless otherwise noted)	YCF sample			
	A	B	C	D
Ascorbic acid	15	15	10	12
Iron	1.2 (FeSO_4_)	1.2 (Fe_2_ lactate)	1.1 (FeSO_4_)	1.2 (FeSO_4_)
FOS	120	Not stated	0	0
GOS	1080	Not stated	500	0
Total FOS and GOS	1200	1200	N/a	0
Fibre (g/100 ml)	0.8	0.8	0.5	0

### Cell culture

The TC7 Caco-2 cell line was used for all experiments (kindly endowed to the Sharp laboratory by Monique Rousset and Edith Brot-Laroche, INSERM U505, [26]); work with this cell line investigating iron uptake and bioavailability has been published in numerous studies, for example [27–29].

Details of the in vitro digestion method have been previously published [27, 28, 30]. Briefly, cells were grown in T75 flasks and seeded into six-well tissue culture plates for experiments. According to the method developed by the Glahn laboratory [30], the in vitro digestions were carried out 13–15 days post-seeding. The media used to feed cells was Dulbecco’s Modified Eagle Medium supplemented with 10% v/v foetal bovine serum, 1% penicillin–streptomycin, 4 mmol/L l-glutamine, 1% non-essential amino acids.

Media were changed to supplemented MEM (10 mmol/L PIPES [piperazine-N, N′-bis (2-ethanesulfonic acid)], 1% antibiotic/antimycotic solution, 11 μmol/L hydrocortisone, 0.87 μmol/L insulin, 0.02 μmol/L sodium selenite (Na_2_SeO_3_), 0.05 μmol/L triiodothyronine and 20 μg/L epidermal growth factor), without foetal bovine serum, to ensure satisfactory cell growth but with low basal media iron levels [30, 31].

### YCF and in vitro digestions

All experiments were carried out with freshly prepared reagents and freshly opened cartons of YCF; only liquid forms of YCF were tested in experiments. 1.8–2 ml of YCF sample was used per digestate depending on sample iron concentration.  

All experiments contained a set of controls: a digest with no added iron to ensure no iron contamination of our system (Blank food digest); a reference digest of 30 μmol/L Fe (Fe alone) equivalent to the iron concentration in YCF digestates; and a positive control digest of 30 μmol/L Fe and 300 μmol/L ascorbic acid (Fe + AA at a 1:10 molar ratio).

The digests were prepared as previously described [8]. Briefly, pepsin was added to the controls or YCF samples to initiate the gastric phase of the in vitro digestion (tested controls or samples herein referred to as digestates), and the pH adjusted to pH 2. As the literature suggests the stomach pH of infants and young children may be higher compared with adults [32], we also conducted a subset of experiments, with a limited number of YCF, in which digestions were initiated with a gastric phase pH of 4. During the gastric phase (at both pHs), digestates were placed in a shaking incubator for 75 min. Subsequently, the pH was raised to 5.0–6.0, bile salts and pancreatin digestive enzymes were added, and the pH was readjusted to 6.9–7.0 followed by another 75 min in the shaking incubator to mimic the small intestinal phase of digestion.

A chamber was created over each individual cell culture well containing the Caco-2 cell monolayer using a 15 kD molecular weight cut-off dialysis membrane fitted over a Transwell insert and held in place with a silicon ring. 1.5 mL of the digestate was then placed in the chamber that was in contact with the cell culture media of the well. The tissue culture plates were placed in a 37 °C incubator for 60 min, while in the incubator they were rotated using a platform-fitted multi-function 3D rotator set at 6 oscillations/min. The inserts were then removed and an additional 1 mL of supplemented MEM added to each well. The cells were returned to the incubator for the following 22 h and then harvested for ferritin.

All YCF samples were tested on a minimum of three separate occasions at gastric phase pH 2,  with *n* = 6 for each treatment. Further in vitro digestions were carried out on one or more occasions with *n* ≥ 6 (specified on figures and tables) for: gastric phase digestion at pH 4; YCF with exogenously added GOS and FOS so that total levels of prebiotics were equivalent across all YCF samples; YCF with exogenously added ascorbic acid so that levels of ascorbic acid were equivalent across all YCF samples; YCF with exogenously added GOS, FOS and ascorbic acid so that levels were equivalent across all YCF; and heat-treated digestates.

### Cell harvest and ferritin analysis

Cells were harvested as previously described [27, 28]. In brief, the monolayers of cells were rinsed and then detached with cell lysis buffer (CelLyticTM—Sigma-Aldrich) and subsequently centrifuged at 6000×*g* for 6 min; supernatant was separated and stored at −80 °C. The samples were analysed for ferritin using the SpectroFerritin MT Enzyme Linked Immunoassay (ELISA; RAMCO, TX, USA). Ferritin levels were adjusted for varying cell numbers/well by measurement of cell protein/well (Pierce Protein BCA assay) and expressed as ng ferritin/mg of cell protein.

### Ascorbic acid analysis

All samples, and GOS and FOS used to fortify Samples C and D to levels equivalent with Samples A and B, were analysed for reduced ascorbic acid (herein referred to rAA) concentrations using the 2,6-Dichloroindophenol Titrimetric method (AOAC 985.33). Briefly, 5 ml of the YCF being analysed, or 5 g of the GOS and FOS powders being tested (prebiotic powders were initially ground in a mortar and pestle with fine sand, then strained), were added to a mixture of glacial acetic acid and metaphosphoric acid, and titrated with a freshly prepared indophenol standard solution. A rAA standard solution was freshly prepared and used to standardise the indophenol titration solution. rAA levels were calculated based on the ascorbic acid equivalent mass titrated to 1 ml of indophenol indicator solution (in relation to the rAA standard solution), adjusted for dilutions factors and the test solution blank titration level.

### Heat treatment

To evaluate the contribution of ascorbic acid to iron uptake, selected YCF digestates, and the iron plus ascorbic acid positive control, were treated for 7.5 min in a 100 **°**C water bath and then plunged into ice, in order to degrade the ascorbic acid [33].

### Statistical analysis

Statistical analysis of the data was performed using GraphPad Prism (version 6.0c GraphPad Software, San Diego, CA, USA). In vitro digestion experiments were analysed using the statistical methods of Motulsky [34]. Data are presented as mean ± SEM and were analysed by one-way ANOVA followed by either Tukey’s multiple comparisons test (all-pairwise across experimental groups) or, where the comparison being made was only between select pairs, Sidak’s multiple comparisons test. Differences between means were considered significant at *p* ≤ 0.05. Ordered increases in data were tested using the nonparametric Jonckheere–Terpstra test. Ordered increases were considered significant at *p* ≤ 0.05. The Jonckheere–Terpstra test was computed in Microsoft Excel. Unless stated otherwise, in the results section the word “significantly” is used to denote statistical significance as indicated by the relevant *p* shown in parenthesis at the end of the statement.

## Results

### Iron bioavailability of Tested YCF at gastric phase pH 2

**Fig. 1:**
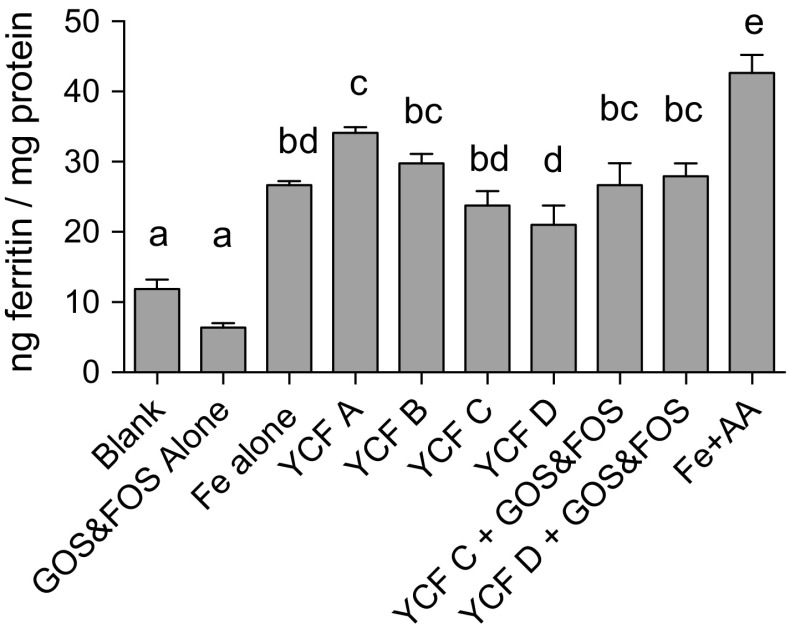
Caco-2 ferritin levels from treatment of cells with digestates of controls, YCF A–D, and YCF C and D supplemented with GOS and FOS to levels equivalent to those found in YCF A and B. Values presented are means of data normalised to the Fe reference control (Fe alone) ± SEM (*n* ≥ 18 for YCF alone, all others *n* ≥ 6). Treatment with YCF A yielded the highest ferritin compared with all other YCF although not statistical when compared to YCF B. The addition of GOS and FOS to YCF C and D increased ferritin to levels statistically equivalent to YCF A and B. Based on an ANOVA (*p* < 0.0001) with Tukey’s multiple comparisons test, post hoc analysis done on an all-pairwise basis; means with *different superscript letters* in a column are statistically different (*p* ≤ 0.05)

Data on iron bioavailability of YCF A–D from the in vitro digestion/Caco-2 cell model are presented in Fig. 1.

YCF A and B, which had the highest levels of prebiotics and ascorbic acid, had the highest iron bioavailability as measured by ferritin formation. YCF A ferritin levels were 43, and 62% greater than YCF C and D, respectively (*p* < 0.0001). YCF B ferritin levels were 25%, greater than YCF C, although this was not statistically significant (*p* = 0.1007), and 41% greater than YCF D (*p* = 0.0113). YCF C and D did not have statistically different ferritin concentrations.

### Iron bioavailability of tested YCF at gastric pH 4

YCF A, B and D (representing the YCF with the highest and lowest bioavailability, respectively) were tested using a gastric phase pH of 4 (Table 2). Changing the pH to 4 did not alter iron bioavailability for any of the tested YCF.

**Table 2:** Ferritin values of Caco-2 cells treated with YCF A, B and D at gastric phase digestions of pH 2 compared with pH4

YCF sample	pH2 ng ferritin/mg protein	pH4 ng ferritin/mg protein	*p* value
YCF A	34.1 ± 0.9	29.9 ± 1.1	0.4239
YCF B	29.7 ± 1.4	30.1 ± 2.5	0.9990
YCF D	21.1 ± 2.8	17.1 ± 3.2	0.5407

Values presented are the mean ± SEM. There was no statistical difference between pH2 and pH4 gastric phase digestions for any of the three samples using Sidak’s multiple comparisons test (*n* ≥ 6)

### Iron bioavailability of YCF to which exogenous GOS and FOS were added

Data on iron bioavailability for these experiments are presented in Fig. 1.

Ferritin levels in YCF C and D were significantly lower than those in YCF A. Ferritin levels of YCF C were 10.25 ng ferritin/mg protein lower than YCF A (30% lower; *p* < 0.0001), and ferritin levels of YCF D were 13.01 ng ferritin/mg protein lower than YCF A (38% lower; *p* < 0.0001). While not statistically significant, ferritin levels of YCF C were also 20% lower than YCF B ferritin levels (*p* = 0.1007). YCF D was significantly lower than YCF B by 29% (*p* = 0.0113). YCF C originally contained 0.5\,g GOS/100 ml milk, whereas YCF D originally contained no supplemental prebiotics. After the addition of a mixture of GOS and FOS to both YCF C and D, so that total prebiotic levels were equivalent to those found in YCF A and B, there was no longer a statistically significant difference between the ferritin levels in any of the four tested YCF although YCF C and D ferritin levels were still 7.42 and 6.15 ng ferritin/mg protein lower (by 22 and 18%), respectively, compared with YCF A.

### Iron bioavailability of YCF relative to ascorbic acid with and without heat treatment

YCF A and B had equal amounts of ascorbic acid according to stated information on the manufacturer’s label (15 mg ascorbic acid/100 ml milk); measured levels were 14.2 and 16.3 mg rAA/100 ml milk, respectively. Ascorbic acid levels in YCF C were reported as 10 mg/100 ml milk (the lowest of all tested YCF) and measured as 10.2 mg/100 ml; YCF D rAA levels were reported as 12 mg/100 ml and measured as 10.1 mg/100 ml.

The reported ascorbic acid/iron molar ratios were 4:1 for YCF A and B and 3:1 for YCF C and D, respectively. Ascorbic acid/iron molar ratios based on measured rAA were: 3.75:1; 4.3:1; 3:1; and 2.7:1; for YCF A, B, C and D, respectively.

**Fig. 2:**
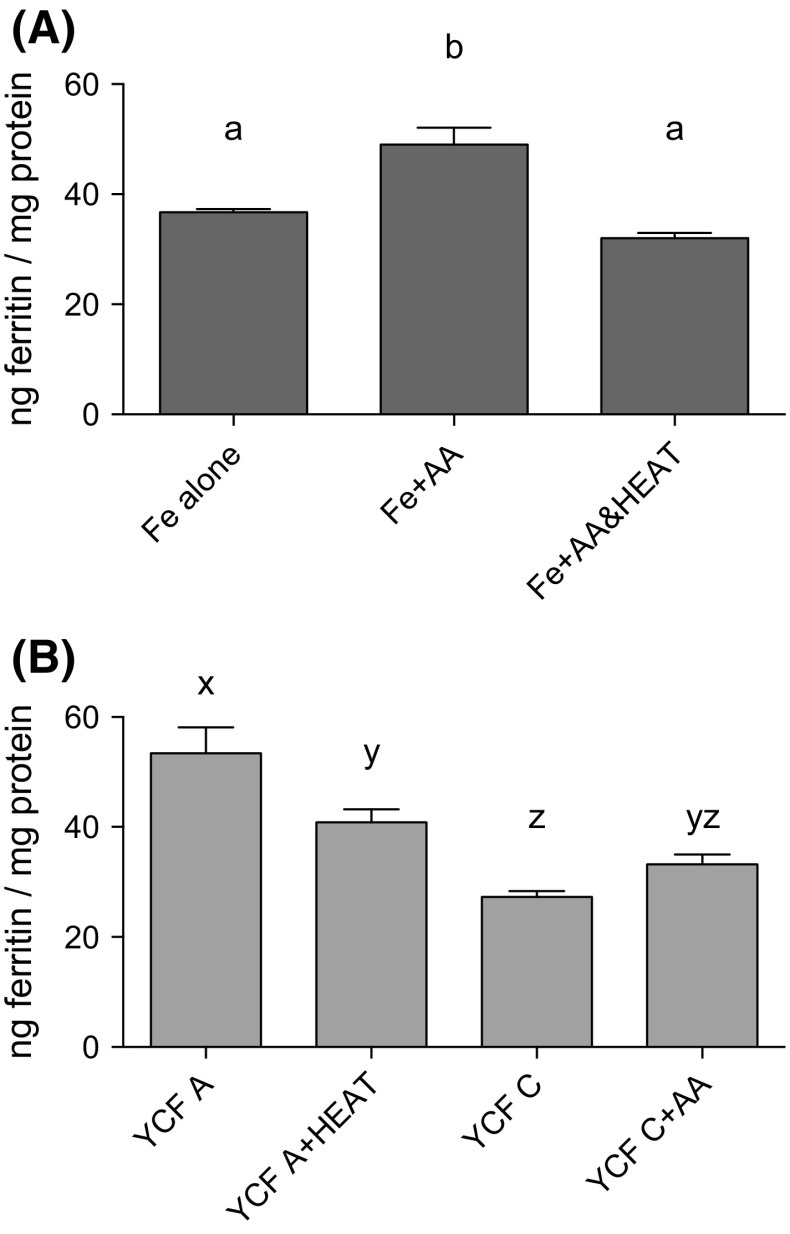
**a** Ferritin levels of Caco-2 cells exposed to control digests, both with, and without, heat treatment to degrade ascorbic acid (AA). Heat treatment of the positive control (Fe + AA at a 1:10 Fe/AA molar ratio) decreased the enhancing effect of AA on iron uptake; ferritin formation was equivalent between the Fe alone and Fe + AA controls. Based on an ANOVA (*p* = 0.0003) with Tukey’s multiple comparison test, post hoc analysis done on an all-pairwise basis; means with *different superscript letters* in a column are statistically different (*p* ≤ 0.0015). **b** Caco-2 ferritin levels of cells treated with YCF A digestates, with, and without, heat treatment, and YCF C digestates, with, and without, added AA (≈1:4 Fe/AA molar ratio). Ferritin levels of YCF A decreased after heat treatment; ferritin levels of YCF C increased after the addition of AA to levels equivalent with YCF A; however, ferritin was still less than non-heat-treated YCF A levels. All presented values are means normalised to the Fe reference control (Fe alone) ± SEM. Based on an ANOVA (*p* < 0.0001) with Tukey’s multiple comparisons test, post hoc analysis done on an all-pairwise basis; means with *different superscript letters* in a column are statistically different (*p* ≤ 0.0074)

In order to ensure that our heat treatment inactivated the ascorbic acid present in YCF samples, we tested whether heat-treating our positive control (Fe + AA) affected ferritin formation. Heat treatment of the positive control significantly decreased ferritin levels compared with the non-heat-treated positive control (*p* = 0.0002); levels were equivalent to those seen in our iron alone controls (i.e. iron added to digestates without any added ascorbic acid; *p* = 0.2227) (Fig. 2a). Heat treatment of YCF A significantly decreased ferritin levels by approximately 24% (from 53.42 to 40.83 ng ferritin/mg protein; *p* = 0.0074); however, YCF A ferritin concentration, even after heat treatment, was still significantly higher by 13.52 ng ferritin/mg protein compared with YCF C levels (the sample with the lowest reported and measured AA levels; *p* = 0.0082). Lastly, we added exogenous ascorbic acid to YCF C to levels equivalent to those found in YCF A; ferritin levels increased by 5.90 ng ferritin/mg protein, or approximately 22%, but were still 20.20 ng ferritin/mg protein less (38%) than those measured in cells treated with YCF A (*p* = 0.0002; Fig. 2b).

### Iron bioavailability of YCF C and D with the addition of exogenous GOS and FOS, with, or without, added ascorbic acid  

**Fig. 3:**
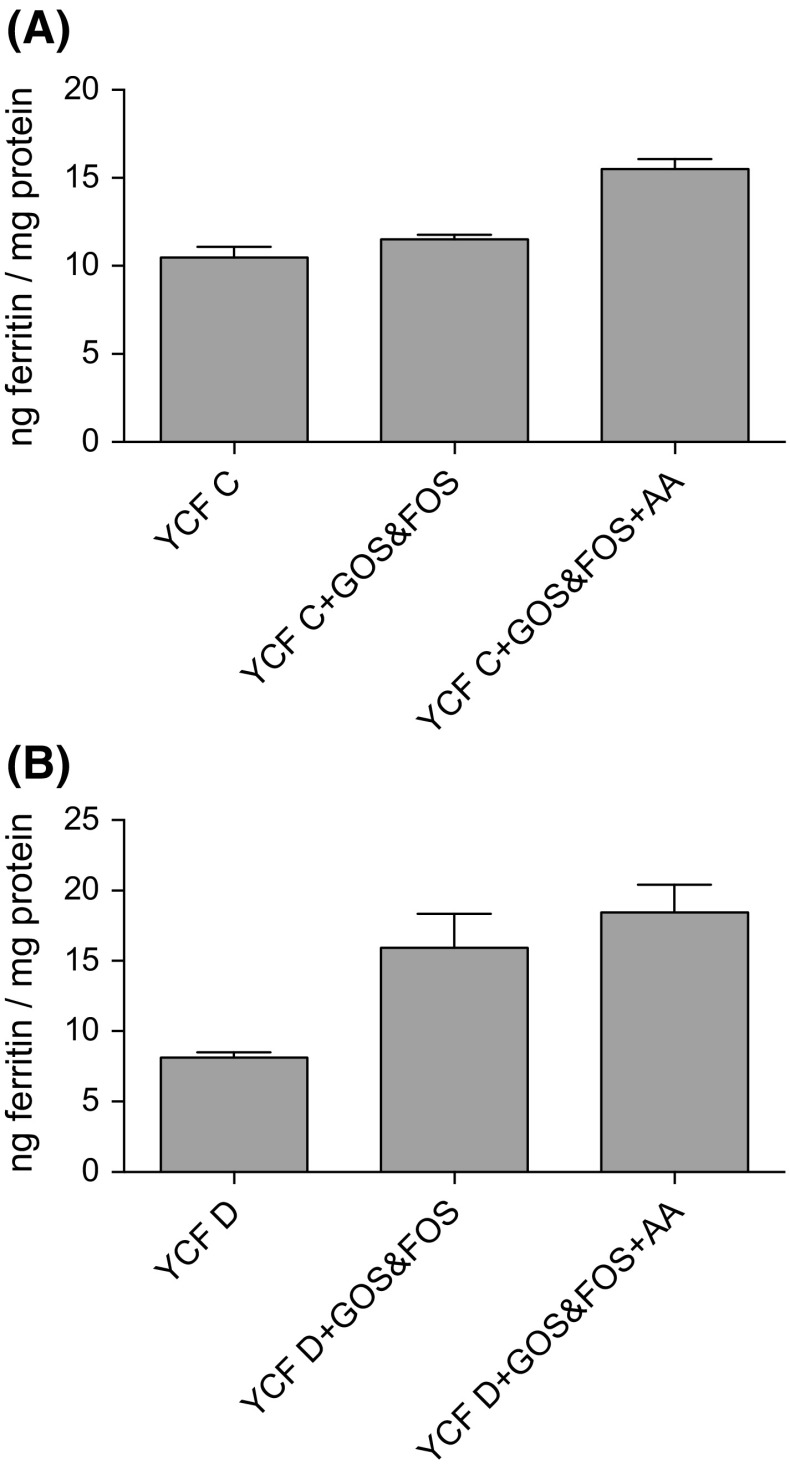
**a** Ferritin levels of Caco-2 cells treated with digests of: YCF C alone; YCF C + GOS and FOS; and YCF C, GOS and FOS, +ascorbic acid (AA, 114 μmol/L; ≈1:4 Fe/AA molar ratio). Increase in ferritin formation was tested using the Jonckheere–Terpstra test and is significant in the order YCF C alone, YCF C + GOS and FOS, and YCF C, GOS and FOS, +AA (*p* = 0.0048). Ferritin levels of Caco-2 cells treated with digests of: YCF D alone; YCF D + GOS and FOS; and YCF D, GOS and FOS, + AA (114 μmol/L; ≈1:4 Fe/AA molar ratio). Increase in ferritin formation was tested using the Jonckheere–Terpstra test and is significant in the order YCF D alone, YCF D + GOS and FOS, and YCF D, GOS and FOS, and AA (*p* = 0.0001)

In order to test whether prebiotics and ascorbic acid had an additive effect on iron bioavailability, we measured ferritin levels of digestates of YCF C and D with added GOS and FOS alone, compared  with digestates of YCF C and D with added GOS and FOS and ascorbic acid. There was an ordered  increase in ferritin formation in the order of digestates of YCF C alone, YCF C and GOS and FOS, and YCF C, GOS and FOS and ascorbic acid (*p* = 0.0048; Fig. 3a); similarly, there was an ordered increase in ferritin formation in the order of digestates of YCF D alone, YCF D and GOS and FOS, and YCF D, GOS and FOS and ascorbic acid (*p* = 0.0001; Fig. 3b).

## Discussion

YCF A and B, with the highest levels of prebiotics and ascorbic acid, had the highest iron bioavailability as measured by Caco-2 cell ferritin formation. YCF A ferritin levels were approximately 1.5 times greater than those of YCF C and D, while YCF B ferritin levels were approximately 1.3 times greater than those of YCF C and D. The addition of prebiotics (a mixture of GOS and FOS) and ascorbic acid to YCF C and D increased iron uptake by approximately 1.5 and 2.3 times, respectively, but neither exogenously added ascorbic acid alone, nor prebiotics alone, elevated ferritin levels to those seen in YCF A, suggesting both factors contributed to the greater iron bioavailability of YCF A.

Previous reports in the literature found that an ascorbic acid to iron molar ration of 4:1 is associated with higher iron bioavailability in high-phytate food products [35]; consistent with this in our study YCF A and B, which had the most bioavailable iron, also had both higher absolute amounts of ascorbic acid and higher ascorbic acid to iron molar ratios compared with the other tested YCF.

Relative to prebiotics, the results of our study are consistent with numerous studies, demonstrating prebiotics enhances iron absorption in rodents and pigs [36–40]. Potential mechanisms driving this increase included: enhanced solubility of iron [40]; a decrease in colonic pH [41, 42]; changes in the profile of the gut microbiota [43]; a direct effect on iron transporter expression in either duodenum or colon [36, 44, 45]; or a systemic decrease in inflammation leading to decreased production of hepcidin, a peptide secreted by the liver in response to inflammation that decreases iron absorption [41]. However, it is not clear whether any of these mechanisms would be relevant in our short-term in vitro studies.

Results from studies evaluating the effects of prebiotics on iron availability carried out in human beings, and also in vitro with the Caco-2 cell line, have been mixed. A recent study in women with low iron status who were given approximately 20 g/d of the FOS-inulin found that although inulin did modify the large bowel microbiome and also lowered colonic pH, there was no influence on iron absorption as measured by the stable isotope technique; iron absorption in the inulin group was 14% higher, but this was not statistically significant [46]. Another study carried out in young men using the same method to assess iron absorption also found no statistically significant effect of inulin, FOS, or GOS on iron absorption, though absorption was some 20% higher in the FOS group compared with control [47]. Both of these studies were relatively small (*n* = 32, *n* = 15, respectively) and therefore may have been underpowered to detect small, but physiologically relevant, increases in iron uptake. Absorption of iron is primarily determined by host iron status, and differences in iron absorption from foods containing less potent enhancers of absorption (e.g. prebiotics) may not be revealed unless some degree of iron deficiency is present, or absolute amounts of bioavailable dietary iron are too low to meet needs. Our results are consistent with a study carried out in infants and young children that demonstrated improved iron status amongst children receiving a mixture of prebiotic oligosaccharides (type not specified) and probiotics [48]. A possible reason for these different study results is that inulin may not be the optimal FOS for increasing iron absorption. Shorter-chain FOS more closely resemble fructose [49], which we have shown increases non-haem iron bioavailability in the Caco-2 cell model [27]. Our previous work demonstrated that fructose added to a ferric iron solution increased levels of ferrous iron by approximately 300% [27], suggesting that the observed increase in iron bioavailability was caused by fructose reducing ferric iron to the more bioavailable ferrous iron. Shorter-chain FOS may be better at improving iron bioavailability compared with longer-chain FOS-inulin if they share some degree of this reducing effect, or secondary to other structural-related differences. In support of this, recent work using GOS suggests that specific structural differences in prebiotics alter their influence on iron absorption [50]. Lastly, human studies evaluating the effects of GOS and FOS given together on iron bioavailability have not, to the authors’ knowledge, been conducted; the two prebiotics together may act synergistically.

Several in vitro studies evaluating the effect of prebiotics on iron uptake have been carried out in the past decade. Two Caco-2 cell-based studies evaluating iron bioavailability from milk- or soy-based yogurts, and common beans, both found that inulin did not improve iron bioavailability from the test food matrix [25, 42]. In contrast, and in agreement with our findings, another in vitro study using iron-fortified cereal biscuits, to which inulin had been added, demonstrated improved iron bioavailability that correlated with increased iron solubility [51]. Both the yogurt and bean-based studies used a higher dose of inulin compared with our study (approximately 17 mg vs. 2.4 mg per digestate) and did not add any GOS; the amount of prebiotics applied to Caco-2 cells in the biscuit study was not specified. In conclusion, the positive results in our present study may relate to several factors: different dose of FOS; different forms of FOS and GOS; and the addition of GOS and FOS together to the tested food matrix.

Young children have the highest risk of ID/IDA amongst pre-adolescent children. The ESPGHAN Committee on Nutrition recommends young children receive a diet rich with iron-containing foods and that excess intake of iron-poor cow’s milk be avoided [3]. However, ID/IDA persists in young children, and thus YCF fortified with iron may be beneficial, as part of a varied diet, in the prevention of ID/IDA. In addition, fortified milk products with the most bioavailable iron would also be predicted to deliver less unabsorbed iron to the young colon. The findings of this study suggest that both prebiotics and ascorbic acid contribute to improved iron bioavailability in YCF. However, ascorbic acid is a relatively unstable micronutrient, and levels may fluctuate depending on storage conditions and timing of use from purchase date [33], whereas prebiotics are relatively stable in foods [52, 53]. Thus, the use of both dietary factors to improve iron bioavailability in fortified milks may be complementary.

Several observational studies in Europe [10, 54] found that intake of YCF was associated with increased dietary intake of iron in young children. A recent cross-sectional study carried out in Western Europe amongst healthy young children found that 11.9 and 3.9% of tested children had ID, and IDA, respectively, and use of cow’s milk as the main milk source was associated with an increased risk of ID [55]. The prevalence of ID in children primarily receiving cow’s milk was 19.7% in contrast with children whose main source of milk was YCF (ID prevalence 5.4%); this difference was highly significant (*p* < 0.001) [55]. The specific YCF used in this study were not specified, nor were study participants’ intakes of ascorbic acid or prebiotics.  

There are several limitations to our study. Although the Caco-2 cell system is a validated model for assessing iron bioavailability including in the food matrixes we tested, the magnitude of effects may not always be accurate [56]. It thus remains for our results to be confirmed in vivo. In addition, we did not analyse the specific forms of GOS and FOS used in this study, and there is evidence that there is structural specificity in terms of the effects of prebiotics on iron bioavailability. Studies looking at specific prebiotic structure and its relation to iron availability would be helpful. Furthermore, although the European Commission regulates the absolute amounts both of prebiotics (and the ratio of GOS to FOS) that may be added to YCF, dose–response analysis of prebiotic effects on iron bioavailability would enable producers to optimise formulation.

In conclusion, use of YCF may contribute significantly to iron intake in young children and thus potentially reduce ID/IDA; however, in the case of iron, the absolute amount ingested does not necessarily reflect the fraction absorbed. Therefore, ensuring that iron in YCF is delivered in a bioavailable form, while minimising the delivery of excess iron to the young gut, would improve the nutritional benefits of YCF. Our results suggest that ascorbic acid and prebiotics in YCF improve iron bioavailability. Further in vivo work to confirm our results and also to explore whether absolute amounts of iron fortification in YCF could be decreased through optimisation of concentration and forms of prebiotics and ascorbic acid warrants investigation.
